# GEROTRANSCENDENCE OF ALASKA NATIVE ELDERS AGING SUCCESSFULLY IN THE ALEUTIAN AND PRIBILOF ISLANDS

**DOI:** 10.1093/geroni/igz038.1908

**Published:** 2019-11-08

**Authors:** Jordan P Lewis, Eric Wortman

**Affiliations:** 1 University of Alaska Anchorage, Anchorage, Alaska, United States; 2 UW School of Medicine, Anchorage, Alaska, United States

## Abstract

Meeting the healthcare needs of Alaska Native (AN) Elders in remote communities is critical to support successful aging and this study allows AN Elders from the Aleutian region to share their experiences and define successful aging, supporting the limited research on AN successful aging. This study interviewed 19 Elders in two communities from the Aleutian region of Alaska. Using a 20-item questionnaire based on Kleinman’s explanatory model to explore successful aging and experiences of being an Elder. Thematic analysis was employed to identify the characteristics and activities of Elders coping and adapting to aging-related changes. This study identified 5 core elements of successful aging, 4 of which formed Lewis’s AN model of successful aging (2011): Mental and Emotional Wellbeing, Spirituality, Purposefulness, and Physical Health and Mobility, and the new element of Gerotranscendence. The unique finding of this study that expands Lewis’s model is the change in mindset Elders experience as they self-reflect. Elders describe being more intentional in their relationships and a stronger connection to traditional cultural and spiritual activities, described by Tornstam (2005) as gerotranscendence. This research will be used locally to develop community specific health promotion and prevention programs to improve Elder services. These findings can also be used by health care providers to help Elders find meaningful activities that promote health and teach individuals to cope with aging-related changes.

